# Crosstalk Between Wnt/β-Catenin and Hedgehog Supports Gli1+ Lineage Osteogenesis in Cranial Sutures

**DOI:** 10.3390/ijms26083508

**Published:** 2025-04-09

**Authors:** Lin Sun, Jie Wang, Shuo Chen, Yang He

**Affiliations:** 1 Department of Oral and Maxillofacial Surgery, Peking University School and Hospital of Stomatology, Beijing 100081, China; sunl9@foxmail.com (L.S.); wangjie_official@163.com (J.W.); 2 National Clinical Research Center for Oral Disease, Beijing 100081, China

**Keywords:** suture, Wnt/β-catenin, Gli1+ stem cells, hedgehog, commitment to osteoblast-lineage cells

## Abstract

Sutures such as fibrous joints in craniofacial bones provide a niche for Gli1+ mesenchymal stem cells (MSCs) in promoting calvarial bone development and growth. However, the underlying molecular mechanism behind the fate of the Wnt/β-catenin regulation of Gli1+ MSCs during calvarial bone formation remains unclear. Here, we showed that β-catenin was colocalized with Gli1+ lineage cells near the osteogenic front within a suture, and postnatal skull development was delayed via a conditional knockout of *Ctnnb1* in Gli1+ MSCs. Calcein–Alizarin Red dual staining revealed that Wnt/β-catenin signal inhibition impaired the rate of bone formation. Furthermore, immunofluorescent staining indicated that Wnt/β-catenin signaling was crucial in facilitating the proliferative capacity of Gli1+ MSCs and their commitment to the osteogenic lineage. Notably, activating hedgehog (Hh) signaling partially restored the suture morphology in *Ctnnb1* knockout mice. Collectively, our findings revealed the crosstalk between Wnt and Hh signaling modulates the fate of Gli1+ MSCs during calvarial bone formation.

## 1. Introduction

Craniofacial bones are flat bones that are distinct from long bones [1,2]. Sutures are fibrous joints in the craniofacial bones comprising a suture mesenchyme surrounded by two osteogenic fronts [3]. Unlike long bones, which are mainly formed via endochondral ossification, craniofacial bones develop through intramembranous ossification. The newly formed intramembranous bone accumulates along the edges of the osteoblastic fronts of the skull [4,5]. In mice, craniofacial sutures are niches for mesenchymal stem cells (MSCs), which support bone development and homeostasis [6,7,8]. Gli1+ cells in the suture mesenchyme are the predominant MSC population in mice and are crucial for the morphogenetic development of craniofacial bones [6,9]. They are strategically positioned in relation to the vascular network and modulated by the Indian hedgehog (Ihh), and they contribute to establishing osteogenic fronts, the periosteum, and the dura [6,10,11].

Craniofacial development involves the intricate spatiotemporal regulation of molecular signaling, and craniofacial deformities occur when this process is disrupted [12]. Craniosynostosis model *Twist1^+/−^* mice showed a diminished Gli1+ cell population [6]. Meanwhile, Gli1+-derived osteoprogenitors are regulated by the mutual interaction between the hedgehog (Hh) signal and bone morphogenetic protein (BMP) signaling, which supports skull bone homeostasis and repair [13,14]. Furthermore, *Axin2^−/−^* mice also exhibited premature cranial suture fusion, a phenotype closely resembling human craniosynostosis [15]. Recently, findings from studies reveal that cells expressing Gli1 and Axin2 are osteogenic stem cells within the human cranial bone niche [16]. Axin2 is predominantly expressed in osteoprogenitor cells and active osteogenic regions, such as the osteogenic front and periosteum. Notably, its expression gradually decreased during the initiation of cranial suture fusion. The functional disruption of Axin2 enhances the differentiation of osteoblasts, potentially accelerating intramembranous ossification and triggering premature suture closure [15]. Based on these findings, Axin2 is a negative regulator of human cranial bone homeostasis. Mechanistically, within the canonical Wnt/β-catenin signaling pathway, Axin2 operates upstream of β-catenin by forming a negative feedback loop that promotes β-catenin degradation, thereby regulating pathway activity [17]. Supporting this regulatory axis of Axin2, Mirando et al. emphasized the critical role of Wnt signaling in cranial morphogenesis and validated the Axin2/β-catenin/cyclin D1 axis as a central regulator of suture mesenchyme development [18]. In their study, they further revealed that cyclin D1, which is directly controlled by β-catenin, is essential for driving the proliferation of skeletal precursor cells during cranial bone morphogenesis.

The proliferation and condensation of suture MSCs and the expression of Hh (*Gli1*, *Ptch1*, and *Ihh*) and Wnt (*Tcf7* and *Wnt10b*) signaling pathway genes were significantly inhibited in cleidocranial dysplasia model *Runx2^+/−^* mice [19]. Mechanistically, Ihh functions as a critical Hh ligand during osteogenic condensation in embryonic mouse calvaria, playing a pivotal role in regulating osteoblast differentiation [20,21]. Ihh deficiency resulted in the downregulation of all osteogenic differentiation markers [21]. *Ihh^−/−^* mice exhibited reduced skull sizes, delayed calvarial ossification, and widened cranial sutures, which were accompanied by the complete absence of HH target genes *Ptch1* and *Gli1* [20]. Complementing these findings, *Ptch1^DL^* homozygous mutants displayed marked hypoplasia or delayed intramembranous ossification in midline-associated frontal, parietal, and interparietal bones, with the persistent malformation of serrated sutures between interparietal bones [22]. It is well established that Wnt/β-catenin signaling is integral in orchestrating the fate, proliferation, migration, polarity, and death of stem cells during their development [23,24]. Deleting β-catenin in Gli1+ cells located beneath the growth plate leads to the osteopenia of cancellous bone in the distal femur, causing a switch toward adipogenesis [25]. However, it is not clearly understood whether Wnt/β-catenin signaling manipulates the proliferative capacity and lineage commitment of Gli1+ MSCs within cranial sutures and how it might modulate calvarial bone development and growth.

To identify the underlying mechanism of Wnt effects on Gli1+ lineage in suture development, we generated *Gli1-Cre^ERT2^;Ctnnb1^fl/fl^* mice and provided evidence that the Wnt/β-catenin inhibition of Gli1+ lineage impaired bone formation during suture development. The in vivo activation of Hh signaling using SAG (Smoothened Agonist) ameliorated the osteogenic differentiation of the Gli1+ lineage and partially rescued sagittal suture development in *Gli1-Cre^ERT2^;Ctnnb1^fl/fl^* mice. Collectively, our findings provide mechanistic insights into the crosstalk between Wnt and Hh signaling in modulating the fate of Gli1+ MSCs during calvarial bone formation.

## 2. Results

### 2.1. β-Catenin Overlaps with Gli1+ Progeny at the Osteogenic Front

**Figure 1:**
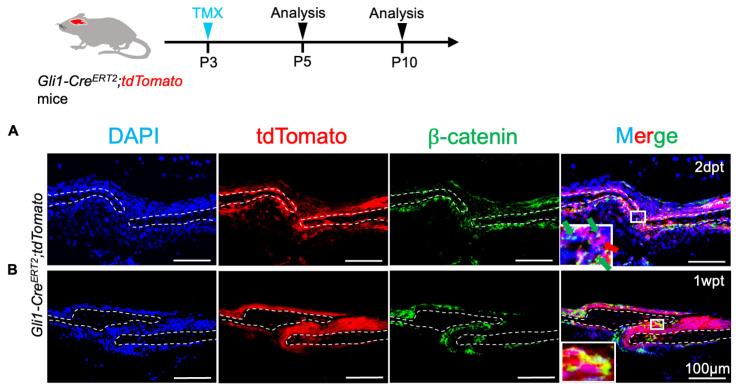
Wnt/β-catenin signaling within the Gli1+ osteoprogenitor lineage of nascent sutures. (**A**,**B**) Immunostaining for β-catenin (green) and visualization of tdTomato (red) within the sagittal suture mesenchyme of *Gli1-Cre^ERT2^*;*tdTomato* mice at 2 days and 1-week post-tamoxifen induction. Red arrow indicates Gli1+ cells; green arrows denote β-catenin cells. Broken lines indicate the outline of the suture. Insets provide a magnified view of the areas outlined in the boxes.

We investigated the expression profile of β-catenin and performed colocalization using the lineage tracing of Gli1+ cells to explore the function of the Wnt/β-catenin signal within Gli1+ cells in manipulating suture development. Following the induction of tamoxifen in 3-day-old mice for 2 days, β-catenin was primarily expressed near the osteogenic front of the cranial suture and was colocalized with a small amount of Gli1+ cells (Figure 1A). However, 1 week after tamoxifen induction, the colocalization of tdTomato with β-catenin near the osteogenic fronts significantly increased, as Gli1-derived cells gradually expanded throughout all sutures (Figure 1B). Thus, the increased overlap of Wnt signaling within the Gli1+ progeny indicates that it may be pivotal for the proliferation of Gli1+ cells, as well as for the transition of osteoprogenitors to osteoblasts.

### 2.2. Loss of Ctnnb1 in Gli1+ Lineage Impairs Craniofacial Bone Formation

**Figure 2:**
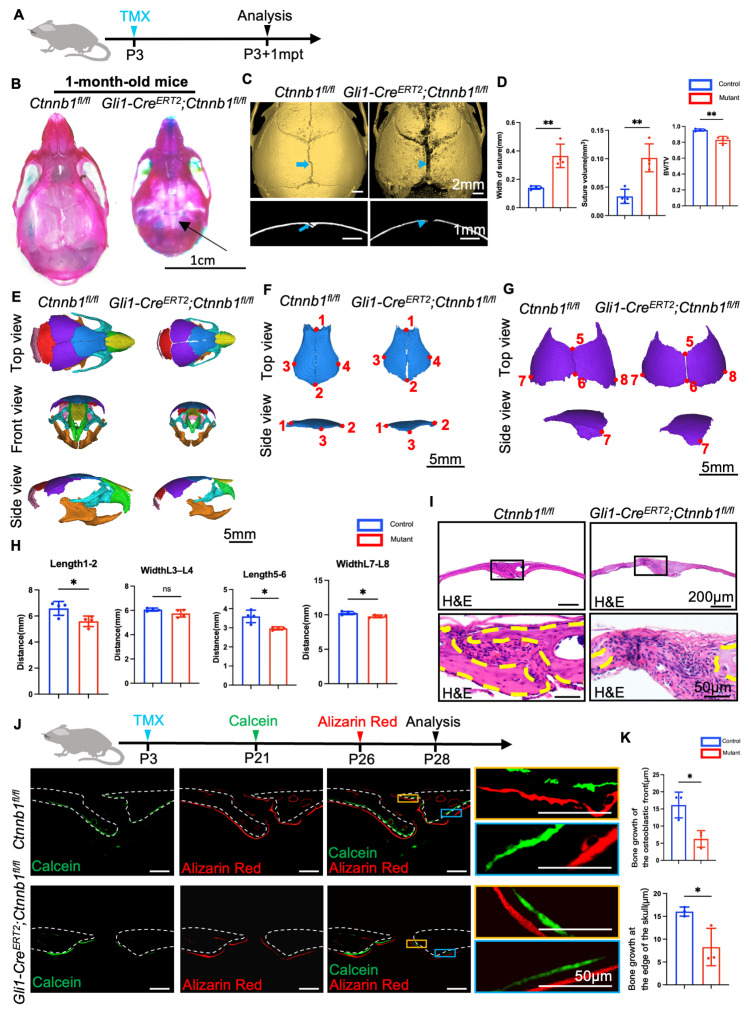
Depletion of *Ctnnb1* in Gli1+ cells induces cranial hypoplasia with the pathological widening of cranial sutures. (**A**) Tamoxifen induction schedule. (**B**) Skulls of *Ctnnb1^fl/fl^* and Gli1-*Cre^ERT2^;Ctnnb1^fl/fl^* mice 1 month after induction, stained with Alcian blue and Alizarin Red S. (**C**) Micro-CT scans of skulls of 1-month-old independent *Ctnnb1^fl/fl^* (control) and *Gli1-Cre^ERT2^;Ctnnb1^fl/fl^* (mutant) mice. Coronal sections of the suture are displayed in the bottom panels. Blue arrows denote the normal morphology of sutures from control mice, while blue arrowheads denote the enlarged suture gap observed in mutant mice. (**D**) Quantitative analysis of width, volume, and bone volume/total volume ratio of sutures from independent *Ctnnb1^fl/fl^* (control) (*n* = 4) and *Gli1-Cre^ERT2^;Ctnnb1^fl/fl^* (mutant) (*n* = 4) mice. (**E**) Three-dimensional reconstruction of craniofacial bones of independent *Ctnnb1^fl/fl^* (control) (*n* = 4) and *Gli1-Cre^ERT2^;Ctnnb1^fl/fl^* (mutant) (*n* = 4) mice. (**F**,**G**) Three-dimensional reconstruction of frontal and parietal bones from four independent *Ctnnb1^fl/fl^* (control) (*n* = 4) and *Gli1-Cre^ERT2^;Ctnnb1^fl/fl^* (mutant) (*n* = 4) mice. (**H**) Quantitative analysis of frontal and parietal bones from *Ctnnb1^fl/fl^* (control) (*n* = 4) and *Gli1-Cre^ERT2^;Ctnnb1^fl/fl^* (mutant) (*n* = 4) mice. (**I**) Hematoxylin and eosin staining of sutures from *Ctnnb1^fl/fl^* (control) and *Gli1-Cre^ERT2^;Ctnnb1^fl/fl^* (mutant) mice 1 mpt (1-month post-tamoxifen induction). The bottom panels show the magnified images. The yellow dashed lines indicate the outline of the suture. (**J**) Illustrative images showcasing the double labeling of calvaria bone with calcein and alizarin red S from 4-week-old *Ctnnb1^fl/fl^* (control) (*n* = 3) and *Gli1-Cre^ERT2^;Ctnnb1^fl/fl^* (mutant) (*n* = 3) mice. Broken lines indicate the outline of the suture. The yellow and blue boxes delineate the areas designated as regions of interest, encompassing the span of the osteoblastic front and the edge of the skull. The boxed areas are enlarged on the right for clearer detail. (**K**) Quantification of mineralizing bone formation. Student’s *t*-tests were performed. Error bars represent the mean ± standard deviation. Statistical significance is denoted as follows: ns = not significant. * *p* < 0.05. ** *p* < 0.01. Four pairs of mice were tested.

We hypothesized that Wnt/β-catenin signaling is vital for suture development, and deleting Wnt/β-catenin within sutures might lead to the dysplasia of cranial sutures. To test our hypothesis, we created *Gli1-Cre^ERT2^;Ctnnb1^fl/fl^* mice, wherein β-catenin depletion occurs specifically in the descendants of the Gli1+ lineage. Physical size analysis revealed that 1 month after tamoxifen induction (Figure 2A), *Gli1-Cre^ERT2^;Ctnnb1^fl/fl^* mice appeared to be notably smaller than those in the control group (Figure S1A). Severe developmental skeletal deformities, including skull hypomineralization and delayed cranial suture development, were evident in 1-month-old *Gli1-Cre^ERT2^;Ctnnb1^fl/fl^* mice observed during Alcian blue and Alizarin Red S (ARS) staining (Figure 2B). The width of the cranial sutures, especially the sagittal sutures, was significantly increased; therefore, the 1-month-old *Gli1-Cre^ERT2^;Ctnnb1^fl/fl^* mice presented with widened sutures. We further used micro-computed tomography (CT) to analyze the craniofacial bones of these mice, and the results showed hypo-ossification, including massive pitted defects of the parietal bone, and obvious morphological deformities, probably due to poorly formed sagittal sutures compared to *Ctnnb1^fl/fl^* mice (Figure 2C). We also analyzed the roots and mandibles. The micro-CT results showed mandible and root hypoplasia compared to control littermates (Figure S1B). Quantitative analyses revealed hypo-ossification in the osteogenic front of the sagittal sutures, as shown by the increased length and volume of the sagittal sutures and the decreased bone volume to total volume ratio (Figure 2D). The root length also decreased (Figure S1C). In addition, we performed a three-dimensional reconstruction of the skull (Figure 2E). The results showed bone defects related to β-catenin loss, including the length of the frontal and parietal bones (Figure 2F–H) and the width of the parietal bone (Figure 2F–H); however, the width of the frontal bone was unaffected (Figure 2F–H). *Gli1-Cre^ERT2^;Ctnnb1^fl/fl^* mice exhibited the delayed eruption of the molars and short mandibles (Figure S1D,E). Thus, these findings indicate that the Wnt/β-catenin inhibition of the Gli1+ lineage could induce severe craniofacial deformity characterized by abnormalities in the skull size and ossification, increased sagittal suture width, and short mandibles and roots.

Craniofacial skeletal development primarily depends on new bone formation [26]. Reduced bone length and low ossification could result from decreased bone formation capacity. To test this, we first performed the histological staining of the sagittal suture and observed that *Gli1-Cre^ERT2^;Ctnnb1^fl/fl^* mice showed much broader sutures, rounded contours of the bone edges, and disordered fibrous structures within the sutures compared to the control group 1 month after tamoxifen induction (Figure 2I and Figure S1F). To assess the influence of Wnt/β-catenin deficiency within the Gli1+ lineage on the capacity of cranial bone formation, we used calcein-ARS double labeling to evaluate the bone formation rates of the skulls in *Gli1-Cre^ERT2^;Ctnnb1^fl/fl^* mice. The distance between the green and red fluorescent labels represented 5 days of new bone formation (Figure 2J). The statistical evaluation showed that new bone formations at both osteogenic fronts and the dura mater of the cranial bones were significantly impaired in these mice (Figure 2K), indicating that the Wnt/β-catenin signal affected the bone formation rate of the osteogenic fronts and the dura mater of the cranial bones.

### 2.3. Knockout of Ctnnb1 in Gli1+ MSCs Impairs Proliferation and Osteoblast Differentiation Activities

**Figure 3:**
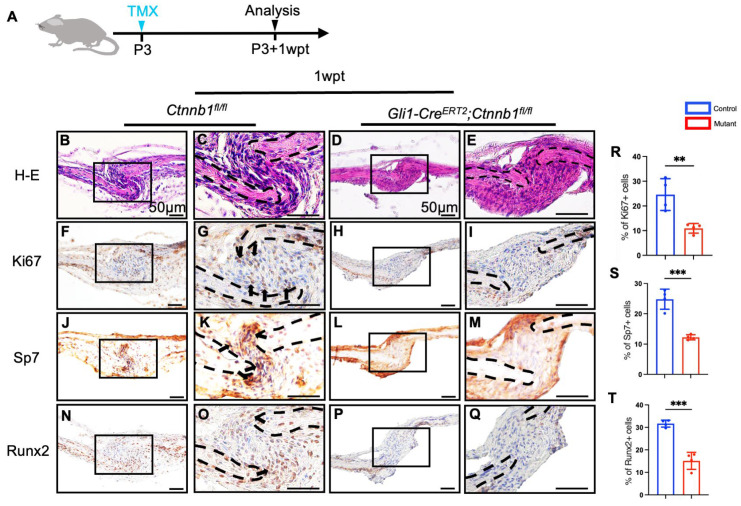
Deletion of β-catenin in Gli1+ lineage leads to weakened proliferation and osteogenic differentiation in *Gli1-Cre^ERT2^;Ctnnb1^fl/fl^* mice. (**A**) Tamoxifen induction schedule. (**B**–**E**) Hematoxylin and eosin staining of sagittal sutures in *Ctnnb1^fl/fl^* (**B**,**C**) and *Gli1-Cre^ERT2^;Ctnnb1^fl/fl^* (**D**,**E**) mice 1-week post-tamoxifen induction. (**F**–**Q**) Immunohistochemical analysis of sutures using anti-Ki67 (**F**–**I**), anti-Sp7 (**J**–**M**), and anti-Runx2 (**N**–**Q**) antibodies. The boxed regions in (**B**,**D**,**F**,**H**,**J**,**L**,**N**,**P**) are magnified in (**C**,**E**,**G**,**I**,**K**,**M**,**O**,**Q**), respectively. Scale bar, 50 µm. (**R**–**T**) Measurement of the proportion of Ki67-positive cells (**R**) in the (**G**,**I**) regions of the sagittal suture, Sp7-positive cells (**S**) in the (**K**,**M**) regions of the sagittal suture, and Runx2-positive cells (**T**) in the (**O**,**Q**) regions of the sagittal suture. Student’s *t*-tests were performed. Four independent samples were used. ** *p* < 0.01. *** *p* < 0.001. Demarcation lines delineate the suture boundaries.

Bone formation is associated with osteoblast differentiation [27]. We investigated whether the damaged skull development in Gli1-*Cre^ERT2^;Ctnnb1^fl/fl^* mice resulted from insufficient osteoblast differentiation. Initially, we performed hematoxylin and eosin (HE) staining of sutures 7 days after tamoxifen induction (Figure 3A). The results showed that Gli1-*Cre^ERT2^;Ctnnb1^fl/fl^* mice presented with poorly formed cranial sutures (Figure 3B–E). To investigate the cellular mechanism underlying the increased suture width, we detected proliferative activity within the sutures and observed decreasing proliferation after the conditional knockout of *Ctnnb1* in Gli1+ lineage cells (Figure 3F–I). Next, we analyzed the differentiation status within the cranial sutures 7 days after tamoxifen induction using osteogenic differentiation markers, including Sp7 [28,29] and Runx2 [30]. Sp7 was robustly expressed in the periosteum, dura, and cranial sutures (Figure 3J). However, within the suture mesenchyme, Sp7 was detected only in a few cells located at the osteogenic fronts and was absent from the mid-suture region (Figure 3K). Meanwhile, its expression was significantly decreased along the osteogenic fronts 7 days after knockout of *Ctnnb1* in Gli1+ MSCs (Figure 3L,M). Moreover, the expression of Runx2 showed a similar pattern and was distinctly downregulated in the sutures of Gli1-*Cre^ERT2^;Ctnnb1^fl/fl^* mice (Figure 3N–Q). Additionally, we quantitatively assessed the expression levels and proliferation of osteogenic markers. The findings revealed that the percentages of Ki67+, Sp7+, and Runx2+ cells within the sutures decreased after *Ctnnb1* deletion in Gli1+ MSCs (Figure 3R–T), consistent with reduced osteogenesis. To further investigate the Wnt/β-catenin signaling regulation of Gli1+-derived progenitor cells along the osteoblast lineage, we co-stained the cranial sutures using immunofluorescence. As osteoblast differentiation progressed, the Runx2+ population within the Gli1-lineage cells decreased near the osteogenic fronts in *Gli1-Cre^ERT2^;Ctnnb1^fl/fl^;tdTomato* mice 1-week post-tamoxifen induction, indicating a marked reduction in osteogenic differentiation capacity in Gli1+-expressing progenitor cells following the lineage-specific knockout of *Ctnnb1* (Figure S2A,B). Collectively, these data show that, within the Gli1+ lineage in the cranial suture, Wnt/β-catenin signaling directly regulated the proliferation and commitment of Gli1+ MSCs to osteogenesis. Additionally, Wnt/β-catenin inhibition within the Gli1+ lineage led to reduced proliferation and osteogenic differentiation activities.

### 2.4. Canonical Wnt Signaling Is Essential for the Activation of Gli1+ Cells Within Sutures

**Figure 4:**
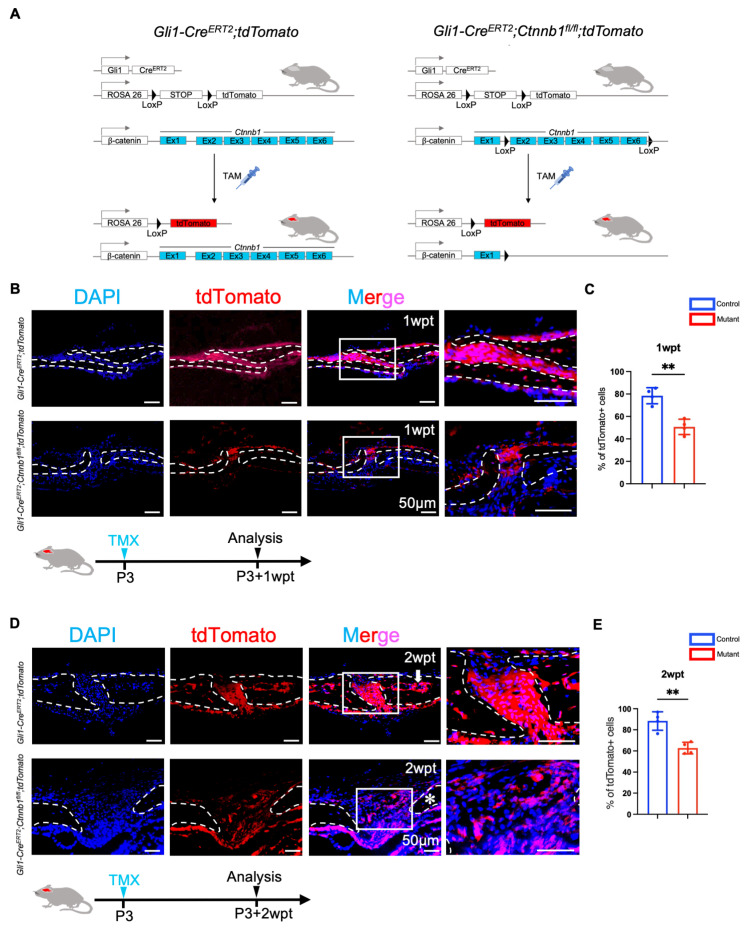
Loss of β-catenin impaired Gli1+ MSC activation. (**A**) Generation of *Gli1-Cre^ERT2^;tdTomato* and *Gli1-Cre^ERT2^;Ctnnb1^fl/fl^;tdTomato* mice. (**B**) Cranial tdTomato immunostaining in the suture mesenchyme from *Gli1-Cre^ERT2^;tdTomato* and *Gli1-Cre^ERT2^;Ctnnb1^fl/fl^;tdTomato* mice 1 week after tamoxifen induction at P3 (Postnatal Day 3). (**C**) Quantitation of the percentage of tdTomato-positive cells within sutures 1-week post-tamoxifen induction at P3 from four independent samples. (**D**) Cranial tdTomato immunostaining in the suture mesenchyme from *Gli1-Cre^ERT2^;tdTomato* mice and *Gli1-Cre*^ERT2^*;Ctnnb1^fl/fl^;tdTomato* mice 2 weeks after tamoxifen induction at P3. tdTomato were not detectable in the marrow cavity of *Gli1-Cre^ERT2^;Ctnnb1^fl/fl^;tdTomato* mice (asterisks). The marrow cavity of *Gli1-Cre^ERT2^;tdTomato* mice (arrow) strongly expressed the marker. (**E**) Quantitation of the proportion of tdTomato-positive cells within sutures 2 weeks after tamoxifen induction at P3. Statistical analyses were conducted using Student’s *t*-tests, and the data are represented as mean ± standard deviation. Four independent samples were used. ** *p* < 0.01. Dashed lines delineate the boundary of sutures.

To understand how β-catenin regulates Gli1+ MSCs during postnatal cranial suture development, we collected suture samples at various time intervals following tamoxifen induction. Initially, we used lineage-tracing analysis to explore the expression profile of Gli1+ within sutures. *Gli1–Cre^ERT2^;tdTomato or Gli1–Cre^ERT2^;Ctnnb1^fl/fl^;tdTomato* mice were treated with tamoxifen 3 days after birth (Figure 4A). At 7 days post-induction, fluorescence-labeled cells were detected throughout the periosteum, dura, and suture mesenchyme, including the osteogenic fronts (Figure 4B). Two weeks later, fluorescence-labeled cells were also found in some osteocytes adjacent to the osteogenic fronts of the cranial bones and within the endosteal compartment of many bone marrow cavities (Figure 4D). However, the number of tdTomato+ cells observed within the sutures of *Gli1–Cre^ERT2^*;*Ctnnb1^fl/fl^*;*tdTomato* mice was considerably low in the bone marrow (Figure 4D), particularly osteocytes. Meanwhile, we performed quantitative analyses and found that the proportion of tdTomato-positive cells within the sutures of *Gli1–Cre^ERT2^*;*Ctnnb1^fl/fl^*;*tdTomato* mice was significantly decreased compared to that in control littermates (Figure 4C,E). Collectively, the loss of Wnt/β-catenin in the Gli1+ lineage profoundly hindered the activation of these cells, consequently leading to a significant blockade in osteogenesis.

### 2.5. Hh Signaling Upregulation Promotes the Activation of Gli1+ Cells and Partially Rescues the Craniofacial Bone Deformity

**Figure 5:**
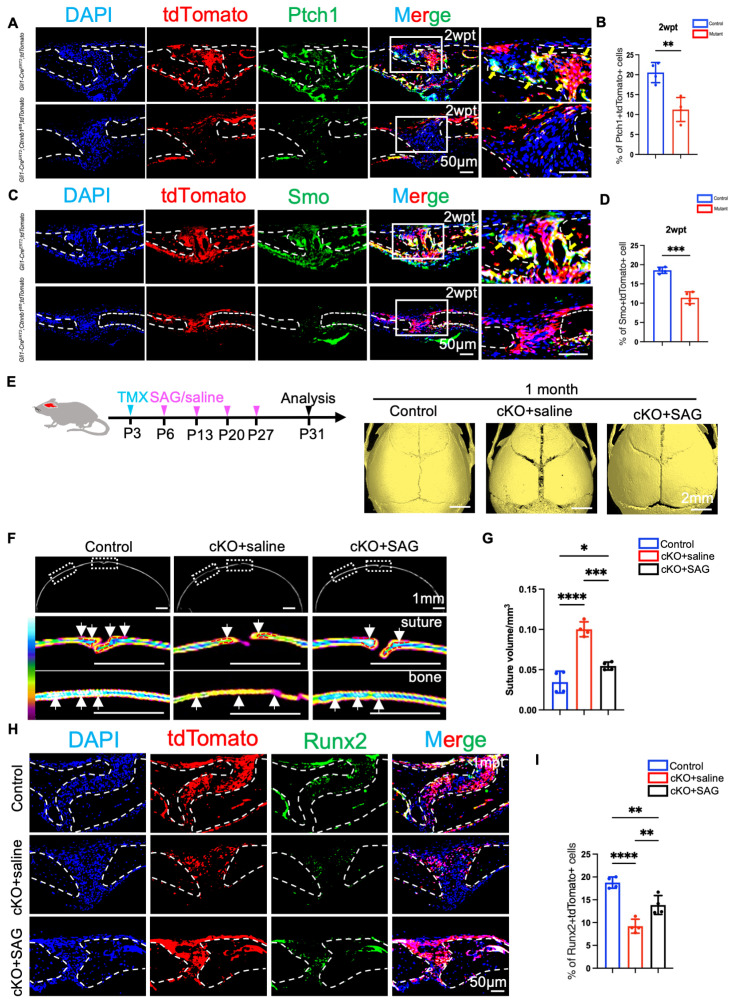
Hh signaling activation partially rescues the craniofacial bone deformity after administrating the Hh signaling agonist. (**A**) Co-immunostaining of Ptch1 and tdTomato in the suture mesenchyme of Gli1-*Cre^ERT2^;tdTomato* and *Gli1-Cre^ERT2^;Ctnnb1^fl/fl^;tdTomato* mice 2 weeks post-tamoxifen induction (2 wpt). Yellow arrows denote colocalization. (**B**) The count of Ptch1-positive and tdTomato-positive cells within the sagittal suture was performed, and the results were expressed as a proportion of the overall mesenchyme cell population in each respective region. (**C**) Co-immunostaining of Smo and tdTomato in the suture mesenchyme of *Gli1-Cre^ERT2^;tdTomato* and *Gli1-Cre^ERT2^;Ctnnb1^fl/fl^;tdTomato* mice 2 wpt. Yellow arrows denote colocalization. (**D**) The count of Smo-positive and tdTomato-positive cells within the sagittal suture was performed, and the results were expressed as a proportion of the overall mesenchyme cell population in each respective region. (**E**) Micro-CT images of skulls from 4-week-old mice of the control, cKO+saline, and cKO+SAG groups. (**F**) The heatmap of micro-CT radiographs showed partially rescued bone density in the *Gli1-Cre^ERT2^;Ctnnb1^fl/fl^;tdTomato* mice (arrow) 1 month after tamoxifen induction at P3 (Postnatal Day 3). The red broken lines indicate the outline of the suture. (**G**) The sagittal suture volume was quantitatively assessed 1 month after tamoxifen induction at P3. (**H**) Co-immunostaining of Runx2 and tdTomato within the sutures of the control, cKO+saline, and cKO+SAG groups. (**I**) The Runx2-positive and tdTomato-positive cells within the sagittal suture were counted, and the results are presented as a proportion of the mesenchyme cell in each region. Student’s *t*-tests or ANOVA was performed. Four independent samples were used. * *p* < 0.05. ** *p* < 0.01. *** *p* < 0.001. **** *p* < 0.0001. Broken lines indicate the outline of the suture.

Gli1 expression may serve as a specific biomarker for suture MSCs or potentially reflect an active physiological response to the activation of the Hh signaling pathway within these cells. The Hh signaling pathway exerts critical control over cellular proliferation, differentiation, and organ morphogenesis [31]. This pathway is initiated when Hh ligands bind to the patched receptor (Ptc), activating Gli transcription through the intermediary actions of the smoothened membrane protein (Smo) [32]. As a critical ligand of the Hh signaling pathway, Ihh localizes precisely at the osteogenic front of cranial sutures and regulates Gli1+ stem cells during osteogenesis. To assess its expression levels, we performed the immunofluorescence staining of the cranial suture of *Gli1–Cre^ERT2^;Ctnnb1^fl/fl^;tdTomato* mice and their control littermates. Ihh expression was inhibited in tdTomato+ cells within the suture mesenchyme of *Gli1–Cre^ERT2^;Ctnnb1^fl/fl^;tdTomato* mice (Figure S3A,B). Furthermore, we observed that Ptch1 and Smo protein expressions were decreased in the suture mesenchyme of *Gli1–Cre^ERT2^;Ctnnb1^fl/fl^;tdTomato* mice compared to the control group (Figure 5A–D), indicating that the loss of β-catenin in Gli1+ lineage cells reduced the Hh signaling pathway. To directly correct the Hh signaling pathway within the sutures, we used SAG, a Smo agonist, to activate Hh signaling. Three days after tamoxifen treatment, *Gli1–Cre^ERT2^;Ctnnb1^fl/fl^;tdTomato* mice were intravenously injected with a vehicle or SAG for 1 month. We found that after induction for 3 days, calvarial bone formation was significantly reduced after the 1-month period in *Gli1-Cre^ERT2^;Ctnnb1^fl/fl^;tdTomato* mice. However, the in vivo injection of the Hh signaling agonist partially rescued craniofacial bone development and suture morphology (Figure 5E). The heatmap of micro-CT radiographs showed that the bone density of the osteogenic fronts and calvarial bone plates were increased in the cKO+SAG group compared to that in the *Gli1–Cre^ERT2^;Ctnnb1^fl/fl^;tdTomato* mutants (Figure 5F). We also quantitatively analyzed the volume of sagittal sutures. The results showed that the SAG group exhibited a significantly increased sagittal suture volume compared to the controls that received the vehicle, demonstrating that the targeted activation of the Hh signaling pathway effectively enhanced new bone formation along the osteoblastic front (Figure 5G). To further confirm the pharmacological rescue of Hh signaling following SAG administration, we analyzed Smo localization using immunofluorescence. Notably, the upregulation of Smo and Gli1+ activation was observed in the sutures of the cKO+SAG group (Figure S4A,B), indicating that the pathway was successfully reactivated. Additionally, immunofluorescence staining showed that in both the suture area and nearby compact bone, the population of tdTomato+ and Runx2+ cells decreased because of *Ctnnb1*’s conditional knockout, whereas SAG injection upregulated the expression levels of tdTomato and Runx2 in these regions (Figure 5H). Intriguingly, we observed that SAG administration failed to obviously increase the expression level of Ki67 among the Gli1+ lineage (Figure S4C,D), indicating that the upregulation of Hh signaling did not significantly affect the proliferation of Gli1+ cells. However, osteogenic differentiation activity in the cKO+SAG group was partially rescued compared to that in the control and cKO groups (Figure 5I). Thus, we concluded that Hh signaling influences the activation and commitment of Gli1 + MSCs to osteoblast-lineage cells, and it could also contribute to the Wnt-mediated development of craniofacial bones (Figure S5A–D).

## 3. Discussion

Bone formation in the cranial suture begins with the condensation and proliferation of mesenchymal stem cells, which serve as precursors of cranial vault bones [5,33,34]. During the whole process of cranial suture development, MSCs are involved in skull growth and development, homeostasis maintenance, and injury repair, all of which are precisely regulated by various signaling molecules [8,35,36,37]. Gli1+ cells are the major MSCs in sutures. In a long bone, the Wnt/β-catenin signal orchestrates the commitment of these cells to the osteoblasts of the Gli1+ lineage [23,25]. However, it is not clear whether Wnt/β-catenin signaling contributes to regulating Gli1+ cells during skull development. In this study, we validated the finding that the Wnt/β-catenin inhibition of Gli1+ cells delays postnatal skull development, and activating the hedgehog signaling pathway could partially rescue the process of craniofacial skeletal development, emphasizing the crosstalk between Wnt and Hh signaling in modulating the fate of Gli1+ MSCs during craniofacial skeletal physiological development.

Wnt/β-catenin signaling orchestrates stem cell pluripotency and determines cellular differentiation fate during developmental processes [23,38]. This intricate developmental cascade integrates signals from various pathways, including fibroblast growth factor, BMP, and Hh, and it is present in various cell types and tissues [23,39]. In prior research, it was confirmed that Wnt/β-catenin signal transduction regulates mouse embryonic osteogenesis downstream of the Hh signal [40,41]. Furthermore, β-catenin mediates the osteoblast differentiation of Gli1+ cells located beneath the growth plate, and its deficiency leads to a shift toward adipogenesis [25]. Gli1+ cells, the main stem cell population within the sutures in mice, are modulated by the Ihh secreted from osteoblast precursor cells [6]. Consequently, it is necessary to explore the underlying mechanism of the Wnt/β-catenin mediated regulation of Gli1+ MSCs in suture development.

Osteogenesis is essential for skeletal development. Gli1+ cells serve as the primary stem cell population within sutures, forming the osteogenic front, periosteum, dura, and craniofacial bones [9,10]. Thus, we hypothesized that the proliferative capacity and differentiation fate of the Gli1+ lineage is modulated by the Wnt/β-catenin signal in the cranial suture because they are in the long bone [25]. We discovered that inhibiting the Wnt/β-catenin signal resulted in the dysplasia of the skull, insufficient bone mineralization, and delayed closure of cranial sutures in our model, indicating that the Wnt/β-catenin signaling pathway is essential for cranial morphogenesis. This result aligns with previous research highlighting that the loss of Axin2, a negative regulator in the Wnt pathway, leads to the premature fusion of cranial sutures, whereas β-catenin promotes the proliferation of skeletal precursor cells by regulating Cyclin D1 [18]. We further verified that the Wnt signaling pathway influences bone formation rates by directly controlling the proliferative capacity of Gli1+ mesenchymal stem cells and osteogenic differentiation. Intriguingly, we observed a phenotype characterized by reduced osteogenesis, along with thin and malformed cranial bones. Furthermore, we explored the potential mechanism of Wnt/β-catenin signal inhibition in the Gli1+ lineage and discovered a significant diminution of osteogenic gene expression at osteogenic frontiers, including Sp7 and Runx2. Sp7 and classical Wnt signaling regulate the direct differentiation of bone progenitor cells into osteoblasts [42]. Runx2, as the upstream gene of Sp7, directly regulates the expression of Sp7 [43,44]. Sp7 acts downstream of Runx2; in osteoblast lineages, Runx2 promotes the differentiation of immature osteoblasts, whereas Sp7 is required for mature osteoblasts to differentiate into osteocytes. Additionally, Wnt/β-catenin signaling acts on preosteoblasts, inducing Sp7 expression, which determines the cell’s commitment to osteoblast differentiation. The colocalization of Gli1+ and Sp7+ cells showed that they were committed to the osteogenic lineage. Therefore, we believe that the Wnt/β-catenin signal could manipulate the fate of Gli1+ MSCs within cranial suture, as well as their commitment to osteoblasts during suture development. Notably, β-catenin deletion hindered the differentiation of Gli1+ cells into the osteogenic lineage, along with a decrease in the expression of key molecules in the Hh signaling pathway (Ptch1, Smo), suggesting that Wnt and Hh signaling may collaborate to maintaining the activity of Gli1+ cells.

The signals of Hh and Wnt interact to regulate bone formation [45]. The Wnt/β-catenin signal functions downstream of Hh to orchestrate osteoblast differentiation, and Hh signaling requires the presence of β-catenin [42]. Using the lineage-tracing technique, we found that Gli1+ cells sequentially generated osteoblasts across all parietal bone locations in postnatal mice. Furthermore, the Wnt inhibition of Gli1+ cells postnatally decreased the number of Gli1+ cells and impaired bone formation. The upregulation of Hh signaling promotes Gli1+ activation and partially rescues sagittal suture morphology. Ptch1 inhibits the Hh signaling pathway, functions as a membrane protein, and is one of the three major transcription factors responsible for activating the Hh signaling pathway [46]. A reduction in Ptch1 expression indicates the downregulation of the Hh signaling pathway. In Gli1+ cells, the conditional knockout of β-catenin followed by treatment with an Hh agonist resulted in slightly increased Ki67 expression, which remained significantly different from the control group. This suggests that the Wnt signaling pathway contributes significantly to regulating the proliferation of Gli1+ cells and their derived osteoprogenitor cells. Consistent with our findings, Zhao et al. used *Gli1–Cre^ERT2^;Smoothened^flox/flox^* mice (*Smo ICKO*) to induce Smo knockout at 1 month of age and found that the loss of Hh signaling had no significant effect on the proportion of proliferating cells in the sagittal suture [6]. Additionally, the in vitro treatment of mouse cranial suture mesenchymal stem cells with ihh did not significantly promote cell proliferation [6]. Furthermore, SAG treatment significantly improved cranial mineralization and osteogenic marker expression; however, the effect was only partially restorative, indicating that Wnt signaling plays an irreplaceable role in regulating Gli1+ MSCs. It is possible that Wnt signaling directly regulates the expression of Cyclin D1 [18], a function that cannot be fully compensated for by Hh signaling. However, the upregulation of the Hh signaling pathway and activation of Gli1+ partially restored cranial morphology and the capacity for osteogenic differentiation. These findings reveal that the Wnt and Hh pathways converge on common downstream targets, such as Runx2, creating a positive feedback loop that synergistically promotes osteogenic differentiation. For example, Runx2, a pivotal transcription factor in osteogenesis, is regulated by both Wnt and Hh signaling pathways [30]. In this study, SAG treatment partially restored Runx2 expression, providing further support for this hypothesis. We demonstrate that Wnt/β-catenin signaling from progenitor cells near the osteoblastic frontier regulates the differentiation fate of Gli1+ MSCs within sutures. In addition, Hh signaling contributes substantially to this ontogenetic process. Furthermore, the significantly reduced the number of Gli1+ cells, resulting in delayed suture closure. The delayed closure could be attributed to an insufficient stem cell supply for generating adequate osteogenic progenitors to stimulate new bone formation in the osteogenic front within the suture.

## 4. Materials and Methods

### 4.1. The Animals

Mouse strains C57BL/6 (JAX#000664), *Gli1-Cre^ERT2^* (JAX#007913), *tdTomato* (JAX#007905), and *Ctnnb1^flox/+^* (JAX#004152) were purchased from Jackson Laboratory. These animals were housed under pathogen-free conditions with a 12 h light–dark cycle. All mice were included in the experimental procedures regardless of sex. All experimental protocols were performed following the ethical standards of the Institutional Animal Care and Use Committee of Peking University (LA2020457).

### 4.2. Tamoxifen Administration

Tamoxifen (T5648, Sigma-Aldrich, St. Louis, MO, USA) was prepared in corn oil (C8267, Sigma-Aldrich, St. Louis, MO, USA) at a concentration of 20 mg/mL. The solution was administered intraperitoneally to the mice at a dose of 1.5 mg/10 g relative to body weight on the third day after birth.

### 4.3. Micro-CT Analysis and Three-Dimensional Reconstruction

Samples were fixed overnight in 4% paraformaldehyde and subsequently preserved in phosphate-buffered saline (PBS) until the samples were ready for scanning. Skulls from *Gli1-Cre^ERT2^;Ctnnb1^fl/fl^* mice and control littermates were subjected to micro-CT analysis (SkyScan 1276, Bruker, Beijing, China) at a 12 μm voxel size. Images in DICOM format were read and displayed in Mimics Medical 19.0, and the three-dimensional craniofacial bone reconstruction was completed according to a previous study [47]. The sagittal suture width was measured as the average of three distinct points along each side of the bone margin: the top, middle, and base.

### 4.4. Alcian Blue and Alizarin Red S Staining of the Skeleton

For the Alcian blue and ARS staining of bones, 4-week-old mice were euthanized, and the skin was carefully removed. After two-time fixation using 95% ethanol (EtOH) overnight, the samples were stained with an Alcian blue solution for 3 days. They were subsequently fixed twice with 70% ethanol, incubated in 95% EtOH overnight, and then treated with 1% KOH for 4 h, followed by incubating in O/N at 4 °C. After staining with an ARS solution for 5 days, the skeleton was cleared using 1% KOH.

### 4.5. Histologic Preparation and Staining

The samples were meticulously excised and subjected to overnight fixation in 4% paraformaldehyde. A 10% EDTA solution (pH 7.4) was used for decalcification, with the treatment durations ranging from 2 to 4 weeks. Subsequently, specimens were embedded in paraffin and microtomed into sections of 6–8 µm in thickness and histologically assessed using H&E and Masson staining, strictly adhering to conventional laboratory protocols.

### 4.6. Immunohistochemical Staining

Paraffin sections, each 6–8 µm thick, were stained using immunohistochemical techniques following the established protocol. The following antibodies were used: Ki67 (1:100, ab15580, Abcam, Cambridge, UK), Sp7 (1:100, ab22552, Abcam, Cambridge, UK) and Runx2 (1:100, A11753, ABclonal, Wuhan, China). The sections were counterstained with hematoxylin. Samples from immunohistochemical staining experiments represent data from four independent experiments.

### 4.7. Immunofluorescence Staining

For cryosections, decalcified samples were subjected to a gradual dehydration process, first in a 15% sucrose solution for 2 h and subsequently transferred to a 30% sucrose solution for an additional 2 h. Next, the samples were further dehydrated in a 30% sucrose/OCT mixture at 4 °C O/N. Specimens were embedded in OCT and microtomed into 10–12 μm thick slices. Immunofluorescence staining was performed following standard procedures. The primary antibody included the Anti–β-catenin antibody (1:100, ab6302, Abcam, Cambridge, UK), Sp7 (1:100, ab22552, Abcam, Cambridge, UK), Ptch1 (1:200, A14772, ABclonal, Wuhan, China), and Smo (1:200, ab236465, Abcam, Cambridge, UK). Alexa Fluor 488 (1:200, ab96899, Abcam, Cambridge, UK) was used as a secondary antibody. 4′,6-diamidino-2-phenylindole (DAPI) was used for counterstaining.

### 4.8. Double Labeling

Calcein (4 mg/mL) was prepared in PBS. Alizarin Red was prepared by dissolving the dye in bacteriostatic water to a precise concentration of 8 mg/mL. Calcein and Alizarin Red S solutions were administered intraperitoneally at a dose of 20 mg/kg body weight, with calcein administered for 7 days and Alizarin Red S administered 2 days before sample collection. Skulls were collected in 70% ethanol. Furthermore, hard tissue sections were prepared by strictly adhering to a previously outlined well-defined protocol [48]. These specimens were sectioned into 20 µm thick slices using a hard-tissue microtome (E300CP, EXAKT, Norderstedt, Germany). The distance between calcein green and alizarin in the red bands was observed using a microscope (BX53, Olympus, Tokyo, Japan) and measured using ImageJ/Fiji 1.53q software.

### 4.9. SAG or Vehicle Treatment

SAG (Selleck, Shanghai, China) was prepared as a stock solution by reconstituting in dimethyl sulfoxide at a concentration of 20 mg/mL. SAG was diluted with 0.9% saline. The mice received intraperitoneal injections of SAG at a dose of 20 μg/g of body weight, which was administered once a week. An equivalent volume of saline was injected intraperitoneally into the mice in the control group at the same frequency.

### 4.10. Quantification and Statistical Evaluation

To determine the proportion of Sp7+, Runx2+, or tdTomato+ cells within the entire cell population, cells within the targeted regions were counted using ImageJ software 1.53q. Statistical analyses were performed using GraphPad Prism version 9, and statistical significance was determined using Student’s *t*-test or one-way ANOVA. The same sample size, N = 4, was used for all experiments except where otherwise stated. Statistical outcomes are expressed as the mean ± standard deviation, with a *p*-value of <0.05 indicating statistically significant differences

## 5. Conclusions

The findings from this study contribute to our understanding of Wnt and Hh signaling within sutures. Our data support the notion that Wntβ-catenin can regulate the proliferation and commitment to the osteoblastic lineage of Gli1+ cells and can also induce the expression of hedgehog pathway genes to regulate the differentiation capacities of Gli1+ MSCs within sutures. However, the downregulation of Gli1+, Sp7, and Runx2 within the suture results in the weaker induction of new bone formations along the osteogenic front, which may be the reason for the delayed closure of the suture. Overall, our findings show that Wnt-dependent Hh signaling modulates Gli1+ MSC differentiation, which is crucial for precisely regulating osteogenesis within the osteogenic fronts and suture development. Future research aimed at elucidating the interactions between these cell types may promote stem-cell-mediated tissue regeneration, which is essential for restoring craniofacial defects.

## Data Availability

Access to all data and materials utilized in this article may be obtained upon request.

## Supplementary Materials

The following supporting information can be downloaded at https://www.mdpi.com/article/10.3390/ijms26083508/s1.

## Abbreviations

The following abbreviations are used in this manuscript:

MSCs	Mesenchymal stem cells;
Hh	Hedgehog;
BMP	Bone morphogenetic protein;
ARE	Alizarin Red S;
CT	Computed tomography;
HE	Hematoxylin and eosin;
Smo	Smoothened membrane protein;
Ptc	Patched receptor.
