# Supplementary material for: Crosstalk Between Wnt/β-Catenin and Hedgehog Supports Gli1+ Lineage Osteogenesis in Cranial Sutures
Source: Int J Mol Sci. 2025 Apr 9;26(8):3508. doi: 10.3390/ijms26083508 (PMC12026649; doi:10.3390/ijms26083508)
Supplement: Supplementary file 1 [file ijms-26-03508-s001.zip › ijms-3479071-supplementary.pdf]

Crosstalk between Wnt/β-catenin and Hedgehog Supports Gli1+ Lineage Osteogenesis in Cranial Sutures

Lin Sun[1,2], Jie Wang[1,2], Shuo Chen[1,2*], Yang He[1,2*]

[1]Department of Oral and Maxillofacial Surgery, Peking University School and Hospital of Stomatology, Beijing 100081, China;

[2]National Clinical Research Center for Oral Disease, Beijing, China

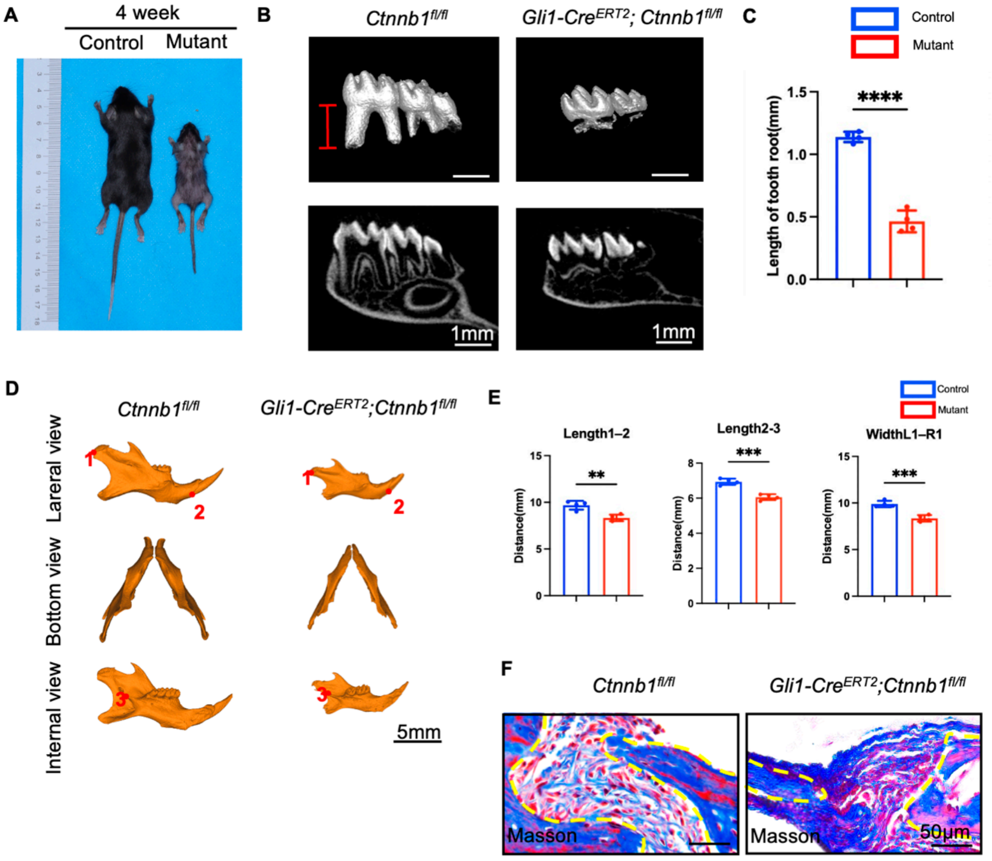

Figure S1.

Phenotypes of *Ctnnb1 cKO* mice. (A) *Ctnnb1*$^{fl/fl}$ (control) and *Gli1-Cre*^{ERT2};*Ctnnb1*$^{fl/fl}$ (mutant) mice were induced at three days of age and analyzed one months later. (B) Micro-CT images of teeth of 4-wk-old independent *Ctnnb1*$^{fl/fl}$ (control) and *Gli1-*

Cre^{ERT2};*Ctnnb1*$^{fl/fl}$ (mutant) mice. (C) Quantitative analysis of length of tooth roots from four independent *Ctnnb1*$^{fl/fl}$ (control) (n = 4), and *Gli1-Cre*^{ERT2};*Ctnnb1*$^{fl/fl}$ (mutant) (n = 4) mice. (D, E) Three-dimensional reconstruction and quantitative analysis of mandible from four independent *Ctnnb1*$^{fl/fl}$ (control) (n = 4), and *Gli1-Cre*^{ERT2};*Ctnnb1*$^{fl/fl}$ (mutant) (n = 4) mice. (F) Masson staining of sutures from *Ctnnb1*$^{fl/fl}$ (control) and *Gli1-Cre*^{ERT2};*Ctnnb1*$^{fl/fl}$ (mutant) mice 1month post-tamoxifen induction (1mpt). Student's t-tests were performed. Four independent samples were performed. **P < 0.01. ***P < 0.001. ****P < 0.0001. The yellow dashed lines delineate the boundary of sutures.

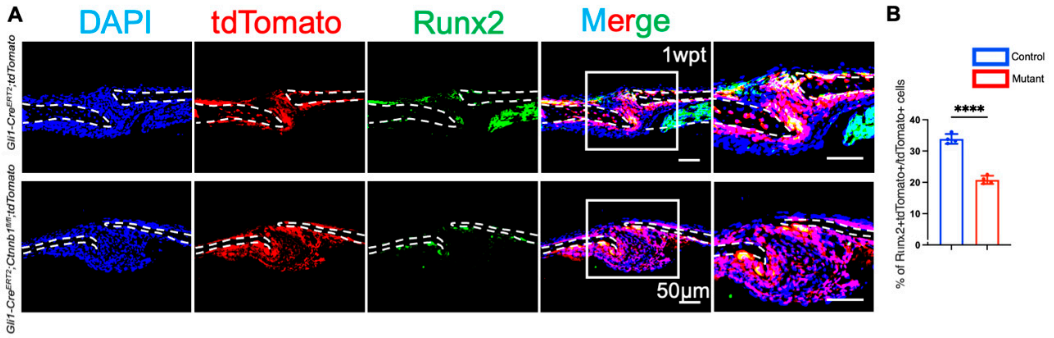

Figure S2.

Knockout of *Ctnnb1* in Gli1+ lineage leads to weakened osteogenic differentiation in *Gli1-Cre*^{ERT2};*Ctnnb1*$^{fl/fl}$;*tdTomato* mice. (A) Co-immunostaining of Runx2 and tdTomato in the suture mesenchyme of *Gli1-Cre*^{ERT2};*tdTomato* mice and *Gli1-Cre*^{ERT2};*Ctnnb1*$^{fl/fl}$;*tdTomato* mice 1 week post-tamoxifen induction (1wpt). (B) The count of Runx2-positive and tdTomato-positive cells within sagittal sutures was performed, and the results were expressed as a proportion of the total tdTomato-positive cells in each respective region. Student's t-tests were performed. Statistical analysis is presented with data represented as mean ± SD. Four independent samples were performed. ****P < 0.0001. Demarcation lines delineate the suture boundaries.

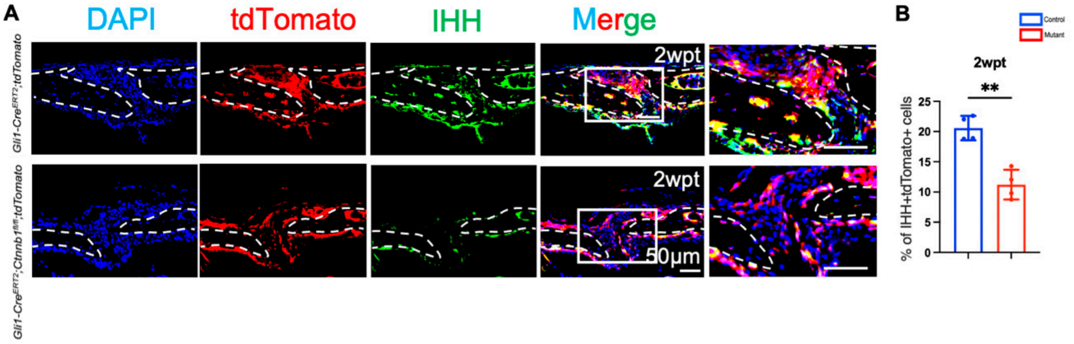

Figure S3.

Downregulation of Hedgehog signaling following knockout of *Ctnnb1* in Gli1+ lineage. (A) Co-immunostaining of IHH and tdTomato in the suture mesenchyme of

Gli1-Cre^{ERT2}*;tdTomato* mice and *Gli1-Cre*^{ERT2}*;Ctnnb1*$^{fl/fl}$*;tdTomato* mice 2 weeks post-tamoxifen induction (2wpt). (B) The count of IHH-positive and tdTomato-positive cells within sagittal sutures was performed, and the results were expressed as a proportion of the overall mesenchyme cell population in each respective region. Student's t-tests were performed. Statistical analysis is presented with data represented as mean ± SD. Four independent samples were performed. **P < 0.01. Dashed lines delineate the boundary of sutures.

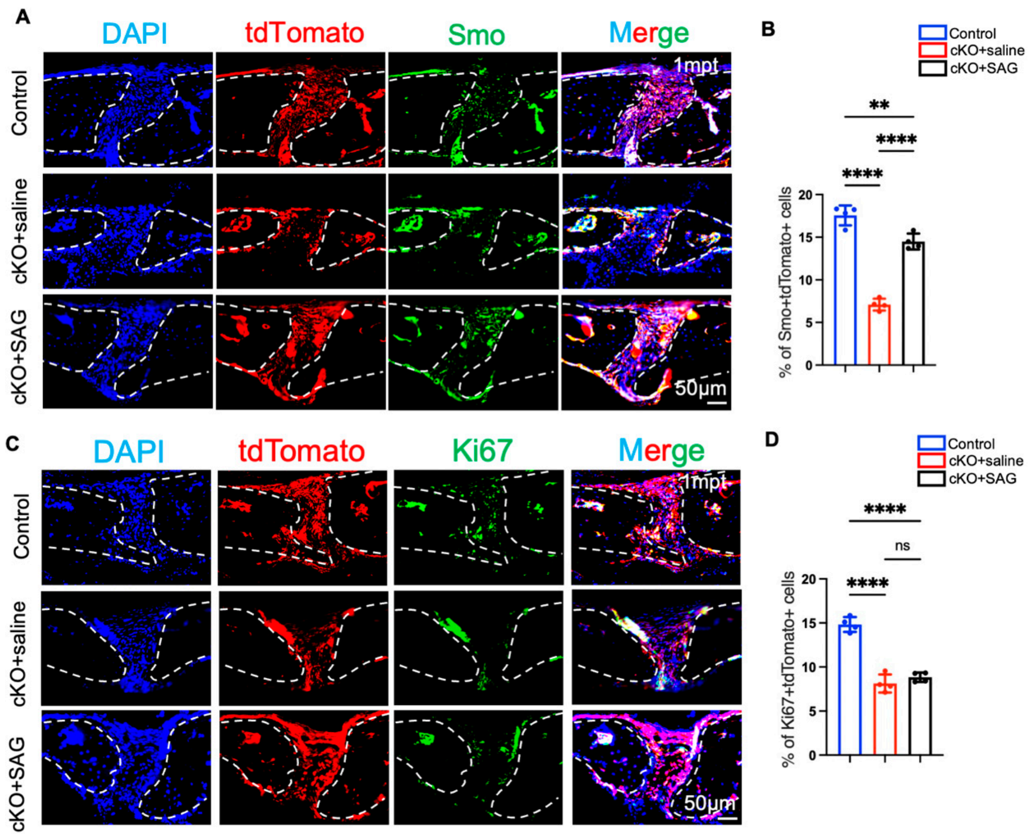

Figure S4.

The activation of Hedgehog signaling following SAG injection. (A) Co-immunostaining of Smo and tdTomato within sutures of control, cKO + saline and cKO + SAG group. (B) The count of Smo-positive and tdTomato-positive cells within the sagittal sutures was conducted and is presented as a proportion of the mesenchyme cell in each region. (C) Co-immunostaining of Ki67 and tdTomato within sutures of control, cKO + saline and cKO + SAG group. (D) The count of Ki67-positive and tdTomato-positive cells within the sagittal sutures was conducted and is presented as a proportion of the mesenchyme cell in each region. ANOVA was performed. Four independent samples were performed. Ns = no significant. **P < 0.01. ****P < 0.0001.

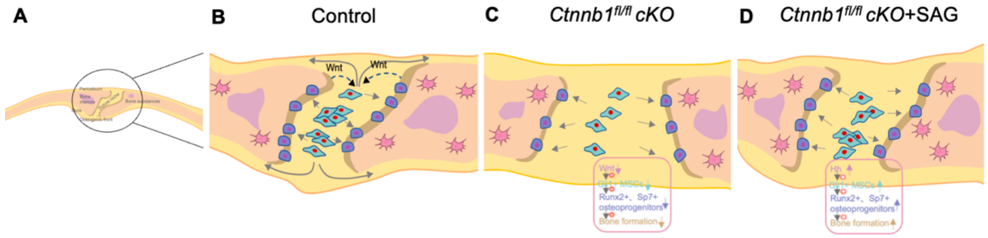

Figure S5.

Diagrams demonstrate Wnt signaling-regulated Gli1+ MSCs is responsible for new bone formation during suture development. (A) Schematic drawing of sagittal suture. (B) Gli1+ MSCs reside in the middle of the suture and are regulated by Wnt/β-catenin signaling from osteogenic fronts, which promotes new bone formation during skull development. (C) Conditional knockout of *Ctnnb1* in Gli1+ MSCs disrupts the activity of Gli1+ MSCs, as well as Sp7+ and Runx2+ osteoprogenitors, thereby diminishing bone formation at the osteogenic fronts. (D) Upregulation of Ihh signaling activates the impaired Gli1+ MSCs, promoting their differentiation into osteoprogenitors cells and subsequently into osteocytes.
